# Timing of the human prenatal antibody response to *Plasmodium falciparum* antigens

**DOI:** 10.1371/journal.pone.0184571

**Published:** 2017-09-26

**Authors:** Samuel Tassi Yunga, Alexander K. Kayatani, Josephine Fogako, Robert J. I. Leke, Rose G. F. Leke, Diane W. Taylor

**Affiliations:** 1 Department of Tropical Medicine, Medical Microbiology and Pharmacology, John A. Burns School of Medicine, University of Hawaii at Manoa, Honolulu, Hawaii, United States of America; 2 The Biotechnology Center, University of Yaoundé 1, Yaoundé, Cameroon; 3 Faculty of Medicine and Biomedical Sciences, University of Yaoundé 1, Yaoundé, Cameroon; Ehime Daigaku, JAPAN

## Abstract

*Plasmodium falciparum* (Pf)-specific T- and B-cell responses may be present at birth; however, when during fetal development antibodies are produced is unknown. Accordingly, cord blood samples from 232 preterm (20–37 weeks of gestation) and 450 term (≥37 weeks) babies were screened for IgM to Pf blood-stage antigens MSP1, MSP2, AMA1, EBA175 and RESA. Overall, 25% [95% CI = 22–28%] of the 682 newborns were positive for IgM to ≥1 Pf antigens with the earliest response occurring at 22 weeks. Interestingly, the odds of being positive for cord blood Pf IgM decreased with gestational age (adjusted OR [95% CI] at 20–31 weeks = 2.55 [1.14–5.85] and at 32–36 weeks = 1.97 [0.92–4.29], with ≥37 weeks as reference); however, preterm and term newborns had similar levels of Pf IgM and recognized a comparable breadth of antigens. Having cord blood Pf IgM was associated with placental malaria (adjusted OR [95% CI] = 2.37 [1.25–4.54]). To determine if *in utero* exposure occurred via transplacental transfer of Pf-IgG immune complexes (IC), IC containing MSP1 and MSP2 were measured in plasma of 242 mother-newborn pairs. Among newborns of IC-positive mothers (77/242), the proportion of cord samples with Pf IC increased with gestational age but was not associated with Pf IgM, suggesting that fetal B cells early in gestation had not been primed by IC. Finally, when cord mononuclear cells from 64 term newborns were cultured *in vitro*, only 11% (7/64) of supernatants had Pf IgM; whereas, 95% (61/64) contained secreted Pf IgG. These data suggest fetal B cells are capable of making *Pf*-specific IgM from early in the second trimester and undergo isotype switching to IgG towards term.

## Introduction

In malaria-endemic regions, the fetus may be exposed to *Plasmodium falciparum* (Pf) antigens (Ags) that cross the placenta. Pf-infected erythrocytes (iE), DNA and/or soluble parasite proteins have been detected in umbilical cord blood samples of <1% to 55% of newborns in sub-Saharan Africa [1,2]. The risk of congenital parasitemia increases with high density of iE in the placental intervillous space (IVS) at delivery [3] and with placental pathology [4,5]. The fetus can also be exposed to Pf Ags in the form of immune complexes (IC) [6,7] that are transferred across the placenta via Fc-neonatal receptors (FcRn) [8]. Both IgG1 and IgG3, the main IgG subclasses produced against Pf Ags [9, 10], are efficiently transported by FcRn [11–13]. Since the IVS is formed by the end of the first trimester [14,15] and FcRn have been detected in human placentas as early as 8–12 wks of gestation [16], fetuses could be exposed to Pf Ags throughout the second and third trimester.

At delivery, newborn lymphocytes exposed *in utero* to Pf can respond to Pf Ags. For example, cord blood mononuclear cells (CBMC) from 25–60% of Kenyan and Cameroonian newborns secreted IFNγ, IL2, IL13, IL4 and/or IL5 in response to stimulation with Pf Ags [17–22]. Not surprisingly, cytokine profiles were altered in cord plasma of Tanzanian neonates born to mothers with placental malaria (PM)-associated anemia [23]. In malaria-endemic areas, newborns have larger spleens compared to newborns in non-endemic areas [24], suggesting splenic lymphocyte proliferation takes place in response to Pf *in utero*. B cell responses also occur *in utero* as Pf-specific IgG and IgM have been detected in supernatants of *in vitro* CBMC cultures [20,25] and in cord plasma, respectively. Since maternal IgM does not cross the placental barrier, Ag-specific IgM in cord plasma is a suitable biomarker for assessing development of the fetal B cell response. Higher total cord plasma IgM levels have been reported in babies born to placental malaria-positive (PM+) compared to PM-negative (PM-) mothers [26], and other studies have detected IgM to an extract of iE and to individual Pf Ags in cord plasma of 2–25% of African newborns [20,22,26–32]. Together, data from previous studies performed predominantly in term newborns, show that the fetus is not immunologically naïve as T and B cell responses can be developed prior to birth. It remains unclear, however, how the fetus responds to Pf Ags at different gestational age (GA) periods.

Development of the human immune system begins during the embryonic period and continues throughout gestation. Hematopoietic precursors are present in the yolk sac and diffuse B lymphocytes have been identified in fetal liver, peripheral blood and spleen by 8–12 wks, 12 wks, and 13–23 wks of gestation, respectively [33]. Primary follicles begin developing in lymph nodes and spleen from 17 and 23 wks, respectively [34], but organized germinal centers that are important in class-switching and affinity maturation, were not detected in spleens of European stillbirths [35,36]. The absence of germinal centers, however, could be due to low antigenic exposure *in utero* in developed countries and does not necessarily imply fetuses cannot make germinal centers. Total lymphocyte counts increase steadily from 20 wks through 40 wks [37] and the B cell receptor repertoire diversifies throughout gestation [38,39]. Thus, the GA at which the fetus is exposed to Ag may influence the resulting antibody (Ab) response.

In the present study, fetal IgM Abs to five Pf Ags were assessed in 232 preterm (GA range, 20–36 wks) and 450 term newborns (≥ 37 wks) in malaria-endemic areas of Cameroon. The influence of GA on the percentage of newborns with Pf-specific IgM, the amount of Pf IgM Ab present, and the number of Ags recognized (breadth) was investigated. Since maternal IC can cross the placenta and potentially stimulate a fetal Ab response, IC containing MSP1 and MSP2 Ags were also measured in a subset of 242 mother-newborn sample pairs and the link between cord IC, PM and GA with cord IgM was examined. Finally, Ab class-switching from Pf-specific IgM to IgG was evaluated using *in vitro* CBMC cultures.

## Materials and methods

### Study population and sample selection

Archival plasma samples and clinical data used were collected between January 1998 and August 2001 at the Central Hospital Maternity in Yaoundé, Cameroon. Yaoundé is a malaria-endemic area with perennial *P*. *falciparum* transmission. At the time of the study, individuals received an estimated 1 or 2 infective mosquito bites per month [40,41]. Since the samples were collected before the implementation of bed net use and intermittent preventive treatment for pregnant women in 2004 [42,43], women most likely were infected several times with Pf during pregnancy and their fetuses could have been exposed to Pf iE and/or Ags.

Preterm and term deliveries were, respectively, defined as births before and after 37 completed weeks of gestation, calculated from the first day of the last menstrual period. Samples from newborns with complete clinical data, and GA at birth ≥20 wks were used, providing a sample size of 682 newborns. Among the 682 babies, there were 40 sets of twins; however, cord blood from only one of each twin was used, so the number of samples (682) equals the number of deliveries. Cord plasma from 100 American newborns whose mothers had not traveled to a malaria-endemic region during pregnancy were obtained from the University of Hawaii Biospecimen Repository and used as negative controls.

For *in vitro* Ab studies, cord blood samples were also collected between July and November 2014 from 64 Cameroonian term newborns. The neonates were born during peak malaria transmission periods in the Nkolbisson (July to October) and Maroua (October to November) health districts of Cameroon. Newborns of HIV-positive mothers were excluded. About 5 mL of blood was drawn from the umbilical vein after clamps had been placed at the sectioned and placental insertion ends of the cord. Blood was collected into EDTA tubes and transported on ice to the laboratory for processing and analysis.

Written informed consent was obtained from mothers of all newborns. Ethical approval for samples collected between 1998 and 2001 was obtained from the Ethics Committee of the Ministry of Public Health, Cameroon, and the Institutional Review Board of Georgetown University, Washington, D.C. Human subject research exemption for use of archival Cameroonian and North American samples was obtained from the Human Studies Program of the University of Hawaii (CHS#21752). The 2014 study was approved by the University of Hawaii Institutional Review Board (CHS#21691) and the Cameroon National Ethics Committee for Human Health Research (No2014/02/416/L/CNERSH/SP).

### Parasitological and hematological studies

Smears of maternal peripheral blood and placental tissue were prepared, stained with Diff-Quick (Baxter Scientific, Inc., Deerfield, IL), and examined for Pf iE by two microscopists. The mother was PM+, if iE were detected in either the peripheral or placental smear or both. Placental parasite density was calculated as the percentage of iE per 2,000 total erythrocytes in the IVS. Maternal anemia was defined as having a packed cell volume (PCV) of less than 30%.

### Testing for admixture of maternal and fetal blood

Polymorphisms at three mini-satellite loci (D1S80, YNZ22 and ApoB) were examined using variable number of tandem repeat (VNTR) PCR assays. Paired maternal peripheral and cord blood samples were evaluated to verify that cord blood was not contaminated with maternal blood cells, i.e., to confirm that Pf IgM antibodies in cord plasma were made by fetal B cells and not by maternal B cells. Mother-stillborn pairs, in whom risk of admixture was presumed to be higher than in mother-livebirth pairs, were selected for the VNTR experiments. To increase sensitivity of maternal DNA detection, DNA was extracted–using the NucleoSpin Blood QuickPure kit, (Macherey-Nagel, Germany)–from cord-blood buffy coat containing a much higher concentration of blood cells than whole blood. About 100 μL of buffy coat was pre-diluted in 100 μL of PBS.

For D1S80, 5′-GAAACTGGCCTCCAAACACTGCCCGCCG-3’ forward primer and 5’GTCTTGTTGGAGATGCACGTGCCCCTTGC-3’ reverse primer were used [44]. For YNZ22, 5’-AGGGAGAGAAAGGTCGAAGAGT-3’ f-primer and 5’-GCCCCATGTATCTTGTGCAGTG-3’r-primer were used [45]. Finally, f-primer 5′-ATGGAAACGGAGAAATTATG-3’ and r-primer 5'-CCTTCTCACTTGGCAAATAC-3’ were used for ApoB [46]. Each 50 μL PCR reaction mix was comprised of 25 μL of 2X GoTaq Green Master Mix (Promega, Madison, WI), 2 μL each of f-primer (100ng/μL) and r-primer (100ng/μL), 4 μL DNA extract and 17 μL of nuclease-free water. The amplification conditions for D1S80 [20], were used for both D1S80 and YNZ22 PCR. ApoB PCR was performed as previously described [46], with the addition of an initial denaturation step at 94°C for 3 min. PCR products were electrophoresed on 2% agarose gel at 5V/cm. For each locus, a mother-newborn pair was informative if the mother possessed two alleles, one of which is not identical to the paternal allele in the newborn. For informative sample pairs, detection of both maternal alleles in the newborn indicated the presence of maternal DNA contamination in cord blood.

### Malarial and control antigens

A panel of malarial Ags was used, including recombinant proteins of the FVO and 3D7 alleles of 42 kDa C-terminal merozoite surface protein-1 (MSP1_42_) expressed in *Escherichia coli*; the FC27 and 3D7 alleles of MSP2 expressed in *E*. *coli*; the 3D7 allele of 83 kDa apical membrane antigen-1 (AMA1) expressed in yeast cells; the 60 kDa conserved region II of erythrocyte binding antigen-175 (EBA175) expressed in yeast cells, and synthetic peptide of the 2.5 kDa ring-infected erythrocyte surface antigen (RESA) containing five EENV repeats. Three control Ags were used, including a recombinant dengue virus non-structural protein (DENV-NS1), dengue virus serotype 2 E protein (DENV2-E) and bovine serum albumin (BSA).

### Coupling of antigens to microplex beads

Each Ag was covalently coupled to microplex polysterene beads (Bio-Rad, USA) with distinct spectral addresses as previously described [47]. To ensure bead surfaces were saturated with Ag, the following amounts of Ag were used per million beads: 1 μg (MSP1-FVO), 1 μg (MSP1-3D7), 0.2 μg (MSP2-FC27), 0.2 μg (MSP2-3D7), 1 μg (AMA1-3D7), 1 μg (EBA175), 10 μg (DENV-NS1), 10 μg (DENV2-E) and 1μg (BSA). Coupled beads were stored in the dark at 4°C at a concentration of 25,000/μL of blocking-storage buffer (PBS pH 7.2, 1% BSA, 0.02% Tween 20, and 0.05% sodium azide).

### Measuring Pf IgM in cord plasma

A bead-based multi-analyte platform assay was used to measure IgM to Pf and control Ags in cord plasma of Cameroonian and North American newborns. Plasma samples were diluted 1:100 in PBS-1% BSA and 50 μL was added to 50 μL of a mixture of equal numbers (3,000 each) of MSP1-FVO, MSP1-3D7, MSP2-FC27, MSP2-3D7, AMA1, EBA175, and RESA-coupled beads in filter plate wells (Millipore, Germany). After 1 h of incubation on a 500-rpm shaker at room temperature (RT) and in the dark, the plates were washed twice with PBS-0.05% Tween 20 and once with PBS-1% BSA. Next, 100 μL of 2 μg/mL phycoerythrin (PE)-conjugated donkey anti-human IgM Ab (Jackson ImmunoResearch, USA) was added to each well and incubated on the shaker for 1 h. After three washes, 100 μL of PBS-1% BSA was added to each well and beads were re-suspended on a shaker for 5 min before analysis in the LiquiChip 100 (Luminex Corp., Austin, TX). Amount of Ab was quantified as the median PE fluorescence intensity (MFI) of 100 beads per spectral address. Cameroonian cord plasma were considered positive for IgM to Pf Ags if the MFI was greater than the mean + 2 standard deviations (SD) of American cord plasma for the corresponding Ag.

To confirm the binding specificity of Pf IgM, 45 Cameroonian cord plasma samples that were positive for MSP1-FVO IgM were tested for IgM to non-malarial antigens that the newborns were unlikely to have been exposed to *in utero*, i.e., BSA, DENV-NS1 and DENV2-E.

### Pf antigen-IgG immune complex assays

IC were measured in peripheral plasma and cord plasma of 242 mother-newborn pairs, including 143 preterm and 99 term deliveries. The paired samples for IC assays were selected based on availability. To capture IC containing malarial Ags, polyclonal anti-MSP1 and anti-MSP2 were used. Polyclonal MSP1 and MSP2 antisera were generated by immunizing rabbits with GST fusion protein containing full length recombinant Pf MSP1 or 2. Abs were further purified by protein G column chromatography and the purity was checked by SDS-PAGE. The Abs were coupled to beads at a concentration of 10 ng of anti-MSP1 and 10 ng of anti-MSP2 per million beads. Coupled beads were incubated for 1 h at RT with a 1:50 dilution of maternal and cord plasma samples. Beads were washed as described above, treated for 1 h with 100 μL of 2 μg/mL of PE-conjugated goat anti-human IgG (Fcγ fragment specific) (Jackson ImmunoResearch, USA) and washed again. The beads were then re-suspended in 100μL of PBS-1% BSA and analyzed in the Liquichip 100. Plasma from 20 American adults were also tested to establish MSP1 and MSP2 IC cut-offs (mean + 2SD).

### CBMC cultures for in vitro IgM and IgG production

CBMC were isolated from freshly-collected cord blood of 64 term newborns by ficoll density gradient centrifugation. Mononuclear cells were harvested, washed three times with 10 mL of 2% Fetal Bovine Serum (FBS) and resuspended at 2 x 10^6^ cells/mL of RPMI-1640 supplemented with 10% Serum Replacement-3 (Sigma-Aldrich), 0.2 M L-glutamine, 0.02 M HEPES, 100 IU/mL penicillin G, 100 μg/mL streptomycin sulfate and 0.1 M sodium pyruvate. Then, 200 μL of CBMC suspension (4 x 10^5^ CBMC)/well was incubated in triplicate wells in 96-well U-bottom plates (Corning, USA) for 5 days at 37°C and 5% CO_2_. Wells containing culture medium only were also incubated for 5 days for comparison. All supernatants were harvested and stored at -80°C until analyzed.

### Measurement of Pf IgM and IgG in culture supernatants

To determine whether Ab-secreting cells in CBMC had isotype-switched *in utero*, culture supernatants were tested for IgM and IgG Abs to Pf Ags. The multiplex assay used was similar to the plasma assay described above, except: 1) Ags were coupled on Magplex magnetic beads (Bio-Rad, USA), 2) culture supernatants were used undiluted, 3) PE-conjugated donkey anti-human IgG (Jackson ImmunoResearch, USA) was used to detect IgG bound to Pf Ags, and 4) the MagPix analyzer (Luminex Corp., Austin, TX) was used to quantify MFIs. The mean + 3SD of MFIs in media controls was used as a conservative cut-off because freshly isolated Pf-naïve CBMC culture controls were not available in Yaoundé.

### Statistics

Fisher’s exact test was used to compare proportions and the Chi-square test for trend was used to identify significant trends in Pf IgM seropositivity rates across GA groups. Confidence intervals (CI) for seropositivity rates were calculated using the modified Wald method. Based on the D’Agostino-Pearson omnibus normality test, many continuous variables exhibited a non-normal distribution prompting the use of non-parametric statistical analyses. Group to group comparisons were done using the Mann-Whitney test and the Wilcoxon signed-rank test for unpaired and paired observations respectively. Changes in the amount of IgM or IC across GA groups were analyzed by the Kruskal-Wallis test including post-test trend analyses. Logistic regression models were used to perform multivariate analyses of factors associated with having Pf IgM in cord plasma, specifically IgM to MSP1 or MSP2. Exposure-related variables, i.e., PM and presence of Pf IC in cord plasma and the timing variable, GA, were included a priori in the model. Maternal anemia was also included in the model because anemia was found to be associated with the breadth of Pf IgM in cord plasma. To further understand if the effect of *in utero* Pf exposure on the odds of having cord IgM was modified by age of the developing fetus, interaction between PM and increasing GA categories was also examined in the regression models.

## Results

### Preterm births

Table 1 summarizes the 682 newborns of the 1998 to 2001 cohort. A total of 232 of the 682 deliveries (34%) were preterm. Having a preterm baby was associated with higher placental parasite density (median percent iE in placental tissue smear [25^th^–75^th^ percentile] was 1.7% [0.6–6.1%] for preterm and 1.0% [0.3–3.4%] for term mothers, p = 0.042), suggesting that maternal malaria contributed to preterm births. Other maternal factors associated with preterm delivery were younger maternal age (24 [21–30] years for preterm; 27 [23–32] years for term, p < 0.001); primigravidity (30.3% of preterm mothers, 22.2% of term, p = 0.025); and maternal anemia (38.4% of preterm mothers, 19.8% of term, p<0.001).

**Table 1:** Descriptive characteristics of the 682 Cameroonian preterm and term newborns whose samples were used for plasma antibody studies.

Characteristics	Preterm (<37 wks)	Term (≥37 wks)	P Value
Number of newborns	232	450	
**Maternal characteristics**			
Age in years	24 [21–30]	27 [23–32]	<0.001
Primigravid women	70 (30.3%)	99 (22.2%)	0.025
Anemia (PCV<30%)	81 (38.4%)	85 (19.8%)	<0.001
Malaria positive	59 (25.7%)	100 (22.2%)	0.339
Placental parasite density in % iE	1.7 [0.6–6.1]	1.0 [0.3–3.4]	0.042
**Fetal characteristics**			
Severity of preterm delivery:			
**1)–Extremely preterm (20–28 wks)**	38 (16.4%)	n/a	
**2)–Very preterm (28–31 wks)**	57 (24.6%)	n/a	
**3)–Moderate to late preterm (32–36 wks)**	137 (59.0%)	n/a	
Live births	194 (83.6%)	443 (98.4%)	<0.001
Twin deliveries	28 (12.3%)	12 (2.7%)	<0.001
Birth weight^Ϯ^ in kg	2.0 [1.5–2.7]	3.3 [3.0–3.7]	<0.001
Low birth weight^Ϯ^ <2500g	137 (68.5%)	35 (8.0%)	<0.001
Placental weight^Ϯ^ in kg	0.5 [0.4–0.6]	0.6 [0.5–0.7]	<0.001

Data are shown as median [25^th^– 75^th^ percentile] or number (%);

^Ϯ^Only singleton deliveries were considered.

P values were calculated using the Mann-Whitney test (medians) and the Fisher’s exact test (proportions). wks, weeks; PCV, packed cell volume; iE, infected erythrocytes; n/a, not applicable; kg, kilogram.

Based on the World Health Organization’s classification of preterm births by clinical severity [48], 16% of the 232 preterm newborns were categorized as extremely preterm (<28 complete wks of gestation), 25% were very preterm (28–31 wks), and 59% were moderate to late preterm (32–36 wks). As expected, preterm singleton newborns had lower birthweights (2.0 [1.5–2.7] kg) than term newborns (3.3 [3.0–3.7] kg), p<0.001. Only 6% (43/682) were stillborn, and clinical information indicated they were not macerated and their placentas were macroscopically intact.

### Confirmation that admixture of fetal and maternal blood had not occurred

Maternal IgM does not cross the placenta and previous studies have demonstrated that maternal blood does not significantly mix with fetal blood during normal vaginal deliveries at term [18,20]. However, 16% of premature newborns were stillbirths (38/232)–in whom risk of blood admixture is theoretically higher than in livebirths–compared to 1.5% (7/450) of term neonates (p<0.001). To evaluate if admixture had occurred, DNA was extracted from peripheral buffy coat of 37 stillbirths and their mothers and examined for polymorphisms at three mini-satellite loci. Of the 37 mother-stillborn pairs that were genotyped, maternal DNA was not detected in 35 cord blood samples (95%) (S1 Fig Gel A and B) and the other two sample pairs were inconclusive as the mother and newborn were either homozygous or had the exact same heterozygous alleles (S1 Fig, Gel C). Because maternal DNA was not detected in any of the stillborn infants, maternal:fetal blood admixture did not have a major influence on the study. Hence, detection of Pf IgM in cord blood indicated that the IgM was produced by fetal B cells.

### Pf IgM in cord plasma

**Fig 1:**
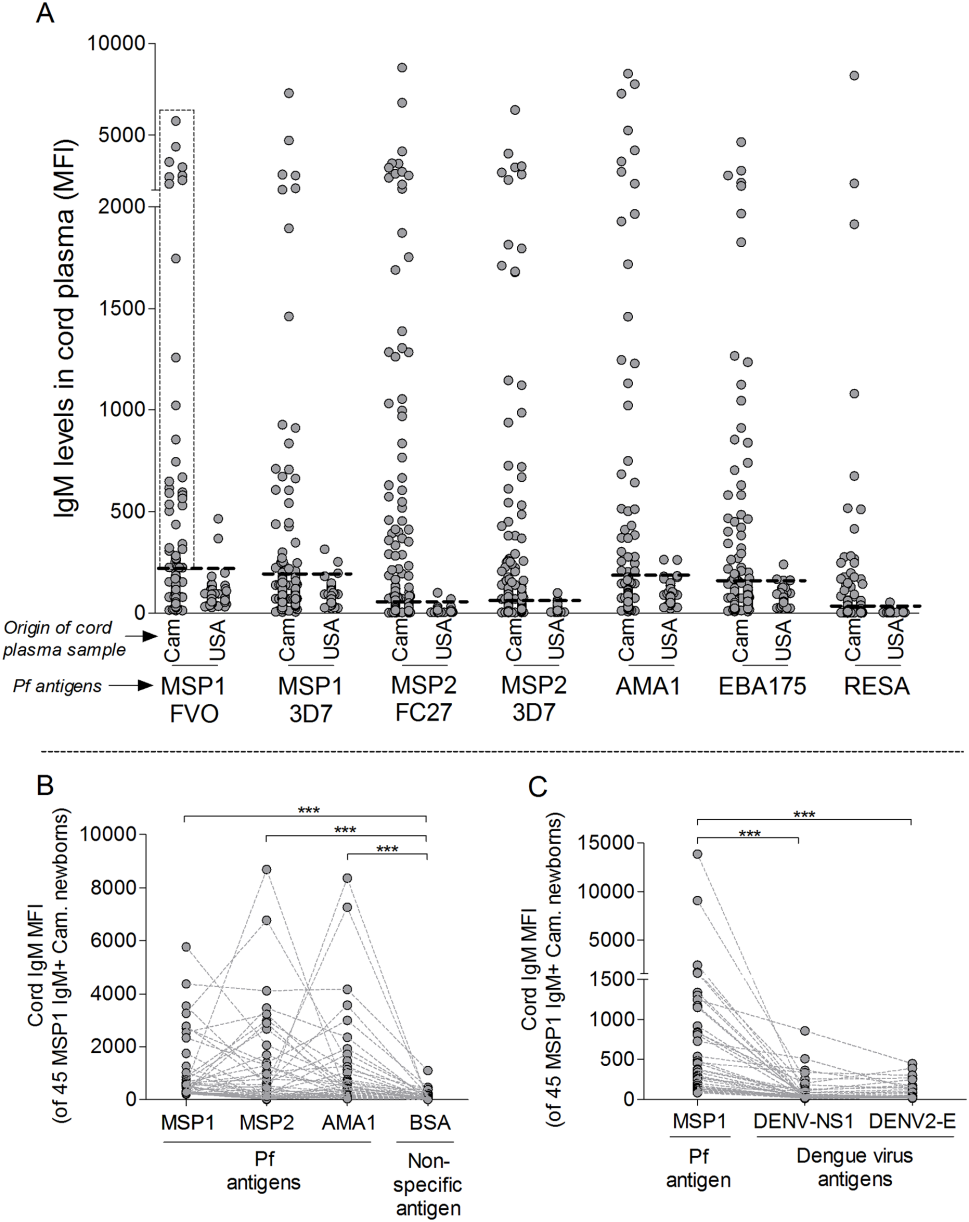
IgM to Pf antigens in Cameroonian and American cord plasma samples. (**A**) Distribution of levels of Pf IgM Abs in 682 Cameroonian (Cam) and 100 American (USA) cord plasma samples. The horizontal dotted lines represent the cut-off for IgM positivity (mean + 2 standard deviations of the USA controls). Only one or two American cord blood samples were above the cut-off for any of the malarial antigens and the samples appeared to be outliers based on the USA MFI distribution. The vertical dotted box within Cam MSP1-FVO shows 45 samples used in Fig 1B and C. The binding specificity of the 45 cord samples positive for IgM to MSP1-FVO was tested for (**B**) BSA and (**C**) two Dengue virus proteins (DENV-NS1 and DENV-2 E). The samples had IgM to Pf Ags but not to the non-malarial Ags. The MFI for a dengue IgM positive control was 4,003 MFI for DENV-NS1 and 847 MFI for DENV2-E (data not shown). Significance of differences in IgM MFI to Pf compared to control Ags was assessed using Wilcoxon signed-rank test (paired comparison): *p<0.01 and ***p<0.0001. MFI = Median Fluorescence Intensity.

As expected, some Cameroonian newborns had IgM to Pf Ags (Fig 1A), indicating that they had been exposed Pf Ags *in utero* and made IgM. Based on the cut-off of mean + 2 SD of 100 American newborns, 7% (95% CI = 5–9%) of Cameroonian newborns were IgM positive for MSP1-FVO, 8% (5–9%) for MSP1-3D7, 18% (15–21%) for MSP2-FC27, 17% (15–21%) for MSP2-3D7, 6% (4–8%) for AMA1, 7% (6–9%) for EBA175 and 9% (7–11%) for RESA. Overall, 169/682, i.e., 25% (22–28%) had IgM to one or more Pf Ags.

To confirm the IgM responses were Pf Ag-specific, cord plasma from 45 newborns who tested positive for IgM to MSP1-FVO were re-screened for IgM to an irrelevant Ag (BSA) and a viral pathogen (DENV) that is uncommon in Yaoundé, Cameroon [49] and does not frequently cross the placenta [50,51] (Fig 1B). Paired comparisons demonstrated that, in the same plasma samples, IgM MFIs were significantly lower for BSA (median [interquartile range] = 26 [10–146]) than for malarial Ags MSP1 (502 [257–938], p <0.0001), MSP2 (290 [62–1,336], p <0.0001) and AMA1 (369 [132–1,076], p <0.0001). IgM MFIs for DENV-NS1 (29 [19–87], p <0.0001) and DENV2-E (34 [21–109], p <0.0001) were also lower than MSP1 IgM. These data show the IgM response to malarial Ags was antigen-specific and not due to cross-reactivity.

### Timing of fetal Pf IgM response to Pf antigens

**Fig 2:**
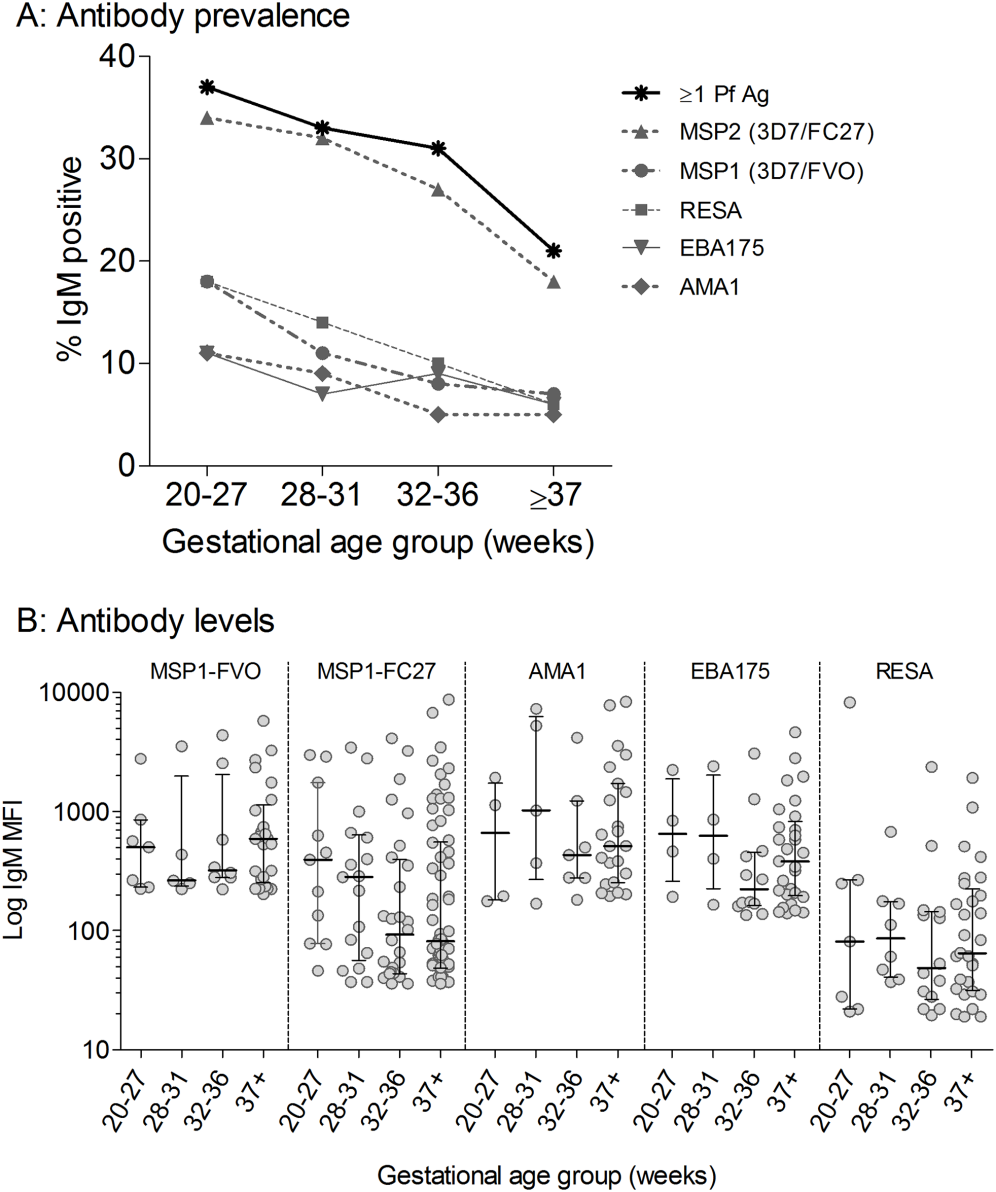
Timing of the prenatal Pf IgM response. (**A**) The percent of newborns (n = 682) positive for IgM to Pf Ags was stratified by gestational age category. The proportion of IgM+ newborns decreased with gestational age, Chi-square test for trend: MSP1 (i.e., positive for IgM to the 3D7 or FVO variant of MSP1), p = 0.008; MSP2 (3D7 or FC27), p = 0.0006; AMA1, ns; EBA175, ns; RESA, p = 0.002; and ≥1 Pf Ag, p = 0.001. For clarity of figures, confidence intervals of proportions are provided in the text but not on the figure. (**B**) Amount of IgM Ab present for newborns who were Ab positive for the specified antigen. Average IgM levels did not differ significantly with GA for any of the antigens (Kruskal-Wallis).

Stratification of the IgM response by GA showed a strong trend for a decrease in the proportion of IgM+ newborns with increasing GA, i.e., the highest percentage of Pf IgM+ were in the extremely preterm, followed by very preterm, moderate to late preterm, and term newborns (Fig 2A). The proportion of newborns who were positive for IgM to ≥1 Pf Ags decreased from 37% (95% CI = 24–53%) at 20–27 wks, to 33% (22–46%) at 28–31 wks, 31% (23–39%) at 32–36 wks and to 21% (17–25%) at ≥37 wks. Despite overlaps in CIs, trend tests showed a significant decrease IgM seropositivity with increasing GA groups (p = 0.001). The trend was also significant for IgM to some individual Pf Ags, including, MSP1 (p = 0.008), MSP2 (p = 0.0006) and RESA (p = 0.002). These results show that B-cell sensitization occurred early in gestation and was not impaired in premature fetuses. The level of IgM to Pf Ags in Ab-positive newborns was comparable in preterm and term newborns (Fig 2B). Average IgM levels did not differ significantly across the different GA groups (Kruskal-Wallis test: MSP1-FVO, p = 0.792; MSP2-FC27, p = 0.187; AMA1, p = 0.766; EBA175, p = 0.409; and RESA, p = 0.848). Therefore, sensitized B cells of preterm and term fetuses produce similar levels of Pf IgM.

**Fig 3:**
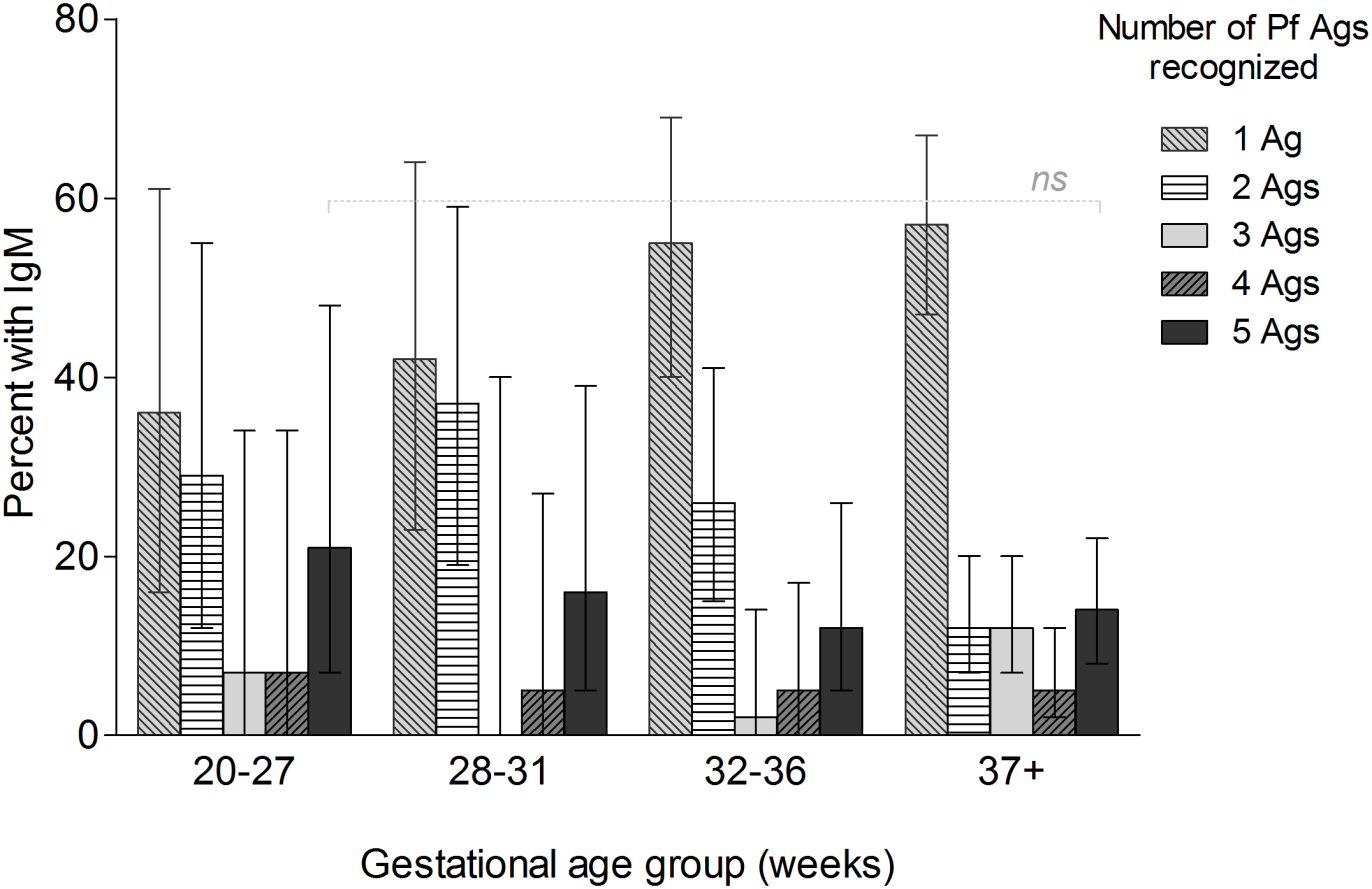
Breadth of Pf IgM response in different gestational age groups. The 169 Cameroonian newborns who were cord IgM+ to ≥ 1 Pf Ag were included, i.e., n = 75 preterm [n = 14 (20–27 weeks), n = 19 (28–31 weeks), n = 42 (32–36 weeks)] and n = 95 term. For each GA group, the bars show the proportion of Ab-positive newborns who produced IgM recognizing 1, 2, 3, 4 and 5 Pf blood-stage Ags. Whiskers represent the 95% confidence interval of proportions. There were no significant (ns) differences in responders to multiple antigens across GA groups.

Since the overall B cell repertoire increases during fetal development, the number of Pf Ags recognized with increasing GA was evaluated (Fig 3). Among the 169 newborns who were IgM+ for ≥1 Pf Ag, 21% (7–48%) of extremely preterm (3/14) and 14% (7–48%) of term newborns (3/94) made IgM to all 5 Pf Ags, including MSP1 (FVO or 3D7), MSP2 (FC27 or 3D7), AMA1, EBA175 and RESA, but the difference was not significant (p = 0.433). No significant trends, for recognizing multiple Pf Ags, were observed across increasing GA groups These findings suggest that the repertoire of B cell clones to Pf Ags was not restricted when the fetuses were exposed to Pf. A significant association was found between maternal anemia and presence of cord IgM to 3–5 Ags (50% were anemic) versus 1–2 Ags (28%) (p = 0.001) (S1 Table).

In summary, these data demonstrate that preterm fetuses can produce similar amount of IgM to as many Pf Ags as term fetuses. Interestingly, the proportion of IgM responders to at least one Pf Ag significantly decreased with GA.

### Timing of fetal exposure to Pf antigen-IgG immune complexes

**Fig 4:**
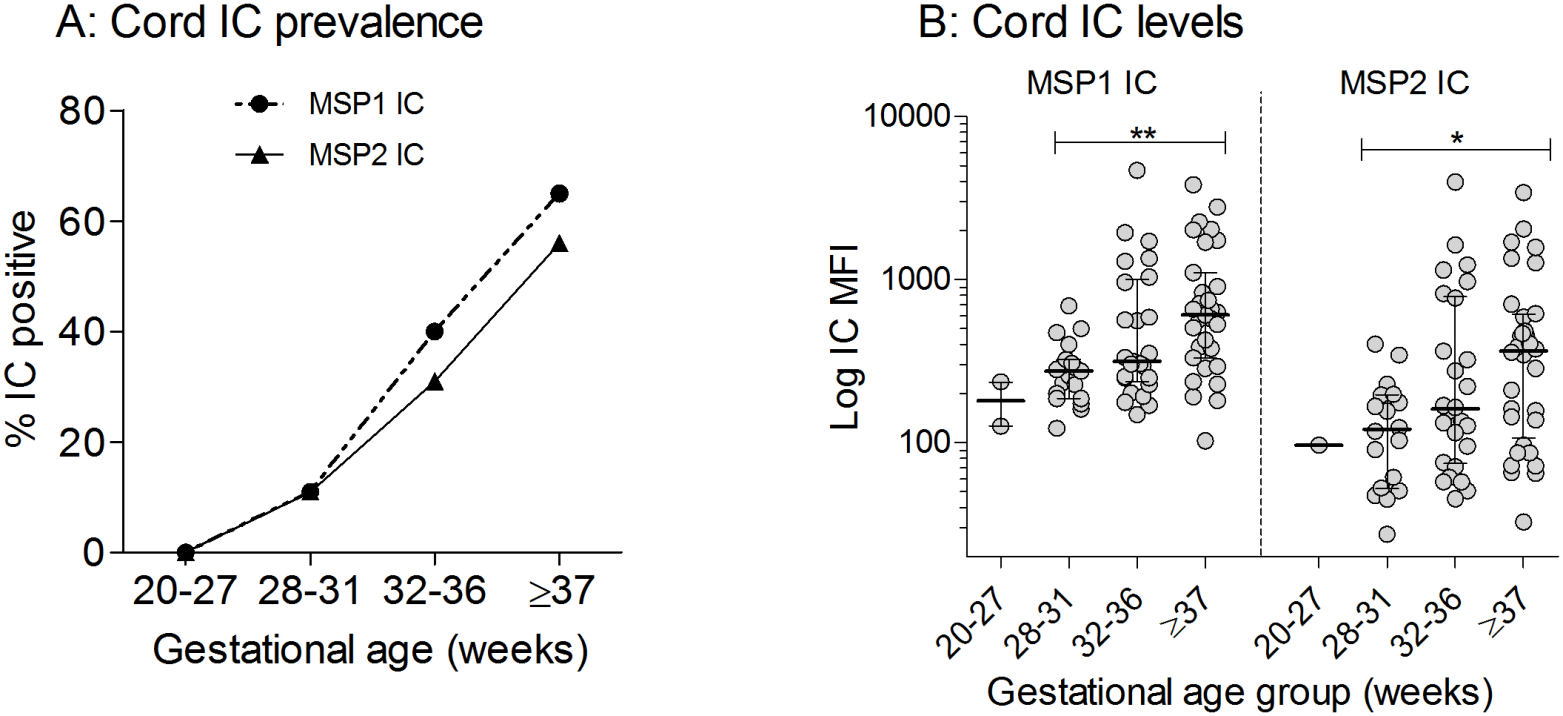
Timing of fetal exposure to maternal immune complexes (IC). Peripheral plasma samples of 77/242 mothers had IC containing MSP1 or MSP2. Cord plasma IC data of the 77 corresponding newborns were analyzed for materno-fetal IC transfer. (**A**) The percentage of cord blood samples with IC increased with GA, especially after 31 wks. (**B**) Average levels of Pf-specific IC in cord plasma. P values were calculated using Kruskal-Wallis test with post-test for trend: *p<0.01 and **p<0.001. The 20–27 wks group was excluded in the analysis because of few (<3) data points.

Plasma samples from 242 mother and neonate pairs were tested for IC containing MSP1 and IC containing MSP2. Overall, 77/242 mothers (32%) had MSP1or MSP2 IC. Since these mothers had the potential of transferring IC to their fetuses, cord IC data from newborns of IC+ mothers were analyzed in relation to GA (Fig 4). Results showed that IC were uncommon in cord plasma prior to 32 weeks of gestation, but rose quickly, such that 65% (95% CI = 47–79%) and 55% (39–72%) of term newborns had IC containing MSP1 and MSP2, respectively (Fig 4A). Likewise, the amount of IC in cord plasma increased with of GA (Fig 4B). For example, the median IC MFI [interquartile range] for MSP1 was 274 [186–323] at 28–31 wks, 314 [238–999] at 22–36 wks and 607 [332–1,103] at ≥37 wks, p = 0.013). Altogether, the GA-related pattern observed for cord IC (Fig 4) was essentially the reverse of that found for cord IgM Ab (Fig 2).

### Factors associated with presence of fetal Pf IgM

To determine which factors were associated with presence of IgM to Pf Ags in cord blood, a logistics model was employed comprising the variables GA, PM status, presence of IC, and maternal anemia (which was associated with breadth of the IgM response) (Table 2). In the adjusted model, odds of being Pf IgM positive were significant in neonates born very prematurely (adjusted OR [95% CI]: 20–31 weeks; 2.55 [1.14–5.85]) and born to PM+ mothers (2.37 [1.25–4.54]). When an interaction between GA and PM status was evaluated, neonates born to PM+ mothers with GA of 20–31 weeks (very premature) had an adjusted risk of 7.87 [2.1–32.2] and those with GA of 32–26 weeks had an adjusted risk of 3.46 [1.02–12.5]. Neither IC nor maternal anemia was associated with cord Pf IgM+.

**Table 2:** Influence of gestational age and placental malaria on production of Pf IgM to MSP1 and MSP2 *in utero*.

Criterion	Subgroup	% of n	Cord IgM (MSP 1 or 2)			
			Crude odds ratio		Adjusted odds ratio	
			OR	95% CI	OR	95% CI
GA, weeks ^a^	20–31^b^	27%	1.76	[0.85–3.62]	**2.55**	[1.14–5.85]
	32–36	32%	1.55	[0.77–3.13]	1.97	[0.92–4.29]
	≥37	41%	Ref.		Ref.	
PM ^a^	Yes	40%	**1.93**	[1.08–3.48]	**2.37**	[1.25–4.54]
	No	60%	Ref			
Cord IC (MSP1 or 2) ^a^	Yes	16%	1.22	[0.55–2.58]	1.48	[0.63–3.35]
	No	84%	Ref		Ref.	
Maternal anemia ^a^	Yes	33%	1.80	[0.99–3.26]	1.48	[0.79–2.75]
	No	67%	Ref		Ref.	
GA:PM interaction ^c^	20–31: PM-yes	7%	**7.93**	[2.21–31.41]	**7.87**	[2.11–32.31]
	32–36: PM-yes	10%	**3.70**	[1.14–12.84]	**3.46**	[1.02–12.45]
	≥37: PM-yes	23%	2.06	[0.74–6.31]	1.92	[0.69–5.94]
	20–31: PM-no	20%	1.79	[0.61–5.65]	1.85	[0.62–5.97]
	32–36: PM-no	22%	1.96	[0.70–6.05]	1.88	[0.66–5.92]
	≥37: PM-no	18%	Ref		Ref.	

^a^ Each variable was adjusted for all other variables (excluding the interaction variable) in the table.

^b^Extremely preterm (20–27 wks, 8% of n) were grouped with very preterm (28-31wks, 18% of n) newborns for multivariate analyses due to small sample size of the 20–27 wks group.

^c^ Interactions between GA groups and PM were adjusted for maternal anemia and cord IC.

Note: OR, odds ratio; CI, confidence interval; Ref., reference subgroup; GA, gestational age; PM, placental malaria; IC, immune complex; n = 242 newborns.

### CBMC of term newborns produce more IgG than IgM

When CBMC from 64 Cameroonian term newborns (see descriptive characteristics of these newborns in S2 Table) were cultured for 5 days and supernatants were tested for both Pf IgM and IgG, supernatants from only 11% (95% CI = 5–21%) contained IgM ≥1 Pf Ag; whereas 95% (86–99%) contained Pf IgG (Table 3). The *in vitro* IgM levels correlated with the plasma IgM levels (MSP1-FVO r = 0.712, p<0.001; MSP2-FC27 r = 0.890, p<0.001; AMA1 r = 0.395, p = 0.002; and EBA175 r = 0.880; p<0.001) supporting the conclusion that IgM in cord plasma was produced by B cells of fetal origin. No significant difference in the proportion of *in vitro* cultures containing Pf IgM or IgG was found between neonates of PM+ and PM–mothers. The finding of Pf IgG responses in newborns of PM–mothers shows that some of the women that were negative for Pf at delivery had been infected during pregnancy and their fetuses were exposed to Pf *in utero*.

**Table 3:** Percent of Cameroonian neonates whose cord blood mononuclear cells produced Pf specific IgM and IgG *in vitro*.

Antigen	Cord plasma IgM			In vitro IgM			In vitro IgG		
	PM neg. n = 24	PM pos. n = 35	All n = 64	PM neg. n = 24	PM pos. n = 35	All n = 64	PM neg. n = 24	PM pos. n = 35	All n = 64
MSP1	0	6	3	0	3	2	67	80	77
MSP2	4	14	9	0	3	2	33	49	45
AMA1	4	17	11	4	3	3	83	80	81
EBA175	4	9	6	8	3	5	50	51	53
RESA	0	9	5	8	11	9	50	63	56
≥1 Pf Ag	8	20	14	12	11	**11**	92	97	**95**

Data are presented as percent of neonates. PM status was not available for 5 mothers. ≥1 Pf Ag = had Ab to one or more tested Ag in cord plasma or CBMC supernatant.

## Discussion

The human fetus develops in an intra-uterine environment with relatively little exposure to foreign Ags. Therefore, little is known about the timing and nature of acquired immune responses during fetal development. Based on mid-20^th^ century observations that fetal mice inoculated with cells from unrelated mouse strains were less likely to reject grafts from the donor strains after birth [52,53], it was originally thought that fetuses recognize all foreign Ags as ‘self’ and become tolerant to them. However, it is clear today that the fetal immune system can recognize and respond to pathogens. Pattern recognition receptors for pathogen-associated molecular patterns have been identified on various fetal cells [54–56] and the fetal inflammatory response syndrome has been describe following microbial invasion of the amniotic cavity [57]. In addition, Ag-specific T and B cell responses have been detected in term newborns exposed *in utero* to Pf [17,19,20,22,27,30–32,25]; to other pathogens including rubella virus, hepatitis B virus, *Toxoplasma gondii*, and *Trypanosoma cruzi* [58–60]; and to inhalant allergens [61,62]. However, data on when the fetus can respond to Ags and produce an adaptive response during development has been difficult to determine.

Malaria during pregnancy presents a unique opportunity to study the timing of fetal immune responses, since: i) women in high transmission areas test positive for Pf in peripheral blood smears an average of 4–5 times during the second and third trimester [63], ii) iE sequester in the IVS leading to placental pathology and sometimes congenital infection [3,5]; iii) sequestration of Pf at the maternal:fetal border can occur from 8–12 wks to term [14,15]; iv) mathematical modeling studies calculate that primigravid women can harbor chronic placental infections throughout pregnancy [64]; and v) Pf Ags can be transported across the placenta as immune complexes [6]. Although not every infective mosquito bite results in PM and transplacental Pf Ag transfer, it is probable that many fetuses in endemic regions become exposed to Pf Ags from the end of the first trimester through term. The present study is the first comprehensive investigation of fetal Ab responses to Pf Ags in a large sample size of neonates (n = 682) with GAs ranging from 20–44 wks, 34% of whom were preterm (<37 wks, n = 232).

Having Pf IgM in cord plasma was strongly associated with PM (Table 2). Overall, 25% (169/682) of Cameroonian newborns, including a 22-wk preterm newborn, were positive for IgM to Pf Ags, Due to the short half-life of IgM, the presence Pf IgM in cord blood suggests a recent *in utero* exposure to Ag. However, IgM in some exposed fetuses may have waned just before delivery, thus the actual prevalence of fetal IgM+ could be higher than 25%. Samples from MSP1 IgM+ newborns did not react with Ags such as BSA and DENV that the newborns were unlikely to be exposed to *in utero* (Fig 1A, 1B and 1C). Since the Ab response was Ag-specific, the IgM Abs had to be produced by conventional B-2 cells and not by B-1 cells that produce ‘innate’ polyreactive IgM. Although ~90% of human cord blood B cells express the mouse B-1cell marker CD5 [65,66], Griffin et al., [67–69] showed that human B-1 cells are CD20^+^CD27^+^CD43^+^CD70^-^ and only comprise about 6% of cord blood B cells. Thus, the potential for B-2 cell responses to Pf develops early in humans.

The ability of the acquired immune system to specifically respond to Ags depends, in part, on the diversity of T and B cell receptors. Rechavi et al., [39] reported an increase in fetal BCR and TCR repertoire diversity with increasing GA. Thus, one might expect preterm newborns to make IgM to fewer Pf Ags than term newborns. Interestingly, the preterm newborns in this study made similar amounts of IgM and recognized as many, if not more, Pf Ags than term newborns (Figs 2B and 3). The actual breadth of Pf IgM in premature and term fetuses may have been underestimated, given that only 5 out of >700 *P*. *falciparum* proteins made by blood-stage parasites [70]) were used to detect Pf IgM in cord blood. Xi et al. [30] reported that cord plasma with high levels of IgM to Pf-infected erythrocytes detected >30 Pf protein bands on Western blots. Thus, it is likely that preterm and term newborns made IgM to additional Pf Ags. GA-related restriction in BCR repertoire diversity does not appear to be the limiting factor in determining the breadth of the fetal Pf IgM response.

Maternal anemia was associated with increased breadth of Pf IgM independently of GA. Maternal anemia has previously been linked with higher placental weight to birth weight ratios [71,72], meaning that the placentas of anemic compared to non-anemic women, have large surface areas. An increase in surface area could translate into an increase in the number of FcRn for IC transport resulting in an increase in transcytosis of diverse IC. Therefore, B cells in fetuses of anemic women may be exposed to more Pf Ags.

Pf IgM was detected in cord plasma from 22 weeks of gestation through term, demonstrating that *in utero* exposure to Pf occurring from this early age can elicit a B-cell response. However, 22 wks may not be the earliest time point because only one newborn in this study was younger (20 wks) and babies born before 20 wks were excluded from the study. Because, Pf iE begin sequestrating in the IVS from ~12 wks and fetal spleens are seeded with B cells over the next 2 months, it is plausible that Pf IgM would be produced by some fetuses aged 14–20 wks.

The proportion of newborns with IgM to Pf Ags decreased with GA (Fig 2A). After adjusting for PM and anemia, early gestation (specifically 20–31 wks) and maternal PM were associated with higher odds of having Pf IgM (Table 2). This implies that for a given equivalent exposure to Pf, fetal B cells at term are less likely to make Pf IgM than B cells at preterm. The term B cells class-switch from making IgM to making IgG as demonstrated by the *in vitro* experiments (Table 3). This finding supports previous reports of class-switch and somatic recombination occurring *in utero* [20,73] and suggests that germinal centers may be formed in fetuses exposed to Pf Ags. It remains to be elucidated if IgG-secreting cells in term newborns are short-lived or long-lived plasma cells and if Pf-memory B cells are generated *in utero*.

The presence of Pf IC in fetal circulation increased with GA (Fig 4), with a rapid increase during the third trimester. This increase corresponds to the period of rapid placental growth and an increase in surface area for transport of nutrients, Ab and other growth factors. Also, IC transfer to the fetus may be influenced by the size of maternal IC. Since preterm mothers had higher parasite density, their IC most likely formed under conditions of relative Ag excess. Such ICs are small, do not fix complement, and are not easily deposited on trophoblasts for transplacental transfer to the fetus. Greater Pf IC deposition occurs in placentas with relatively low parasite densities [74]. In the literature, the relevance of ICs in eliciting immune responses in human and animal hosts is contentiously debated, as ICs can enhance Ag processing and T-cell stimulation or suppress responses by masking Ags and blocking T-cell/B-cell interactions or exert no effect at all [75]. Since Pf IgM occurred early and transfer of IC later in gestation, Pf IC may play a small role in that initial *in utero* priming if naïve B cells.

**Fig 5:**
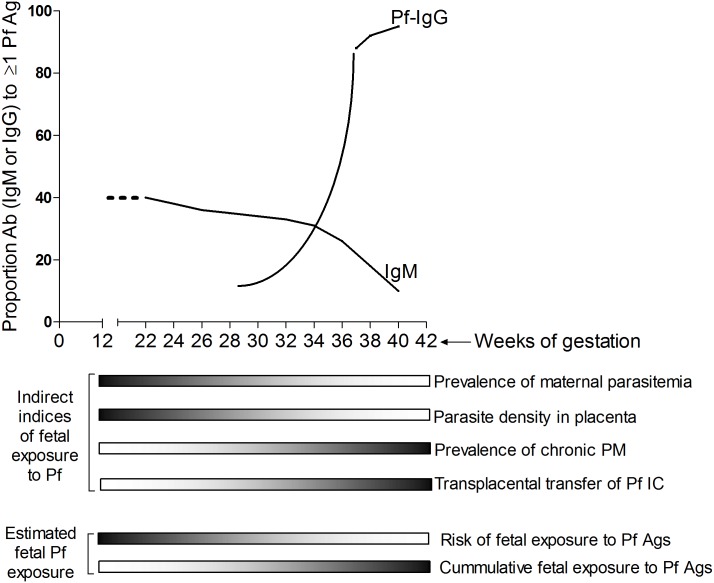
Model for the timing of fetal exposure and Ab responses to Pf Ags. The model is an estimate of the likelihood of fetal exposure, and fetal Ab response, to Pf through the second and third trimesters of gestation. On the horizontal gray scales, dark shade = high; and light shade = low. Infected erythrocytes can sequester in the placenta from ~12 wks of gestation when the IVS are formed. The incidence of maternal malaria and the density of Pf parasites in the IVS are high during the second trimester, thus increasing the risk of exposure to Pf in utero. At this time of gestation, the fetus is already capable of making Pf-specific IgM. As gestation advances, fetal B cells receive secondary exposures to Pf Ags and class-switching to Pf IgG production occurs. The proportion of fetuses with Pf IgM drops rapidly at term while the proportion of Pf IgG responders increases to almost 95% at 40 weeks.

In conclusion, we propose a model for the timing of fetal exposure and Ab responses to Pf (Fig 5). The fetus may be exposed more than once to Pf Ags, from early in the second trimester through term. In response to Pf Ags, both preterm (including extremely preterm) and term fetuses are capable of producing Abs that specifically recognize Pf Ags; however, the Ab response during the second trimester are predominantly IgM while term fetuses predominantly make IgG to Pf Ags.

## Supporting information

S1 Fig Genotyping of VNTR loci for detection of maternal blood admixture with cord blood. Bands represent peripheral maternal blood (M) and cord blood (C) VNTR alleles for mother-stillborn sample pairs (SP). Eight SPs (out of 37 SPs analyzed) are represented. **Gel A** shows D1S80 alleles in SP1through SP7. Maternal DNA was not detected in cord blood compartments of SP1, 2, 3, 4 and 5. SP6 and SP7 were not informative at the D1S80 locus since the mothers were homozygous. Gel B shows YNZ22 alleles for the same SPs. SP1, 2, 3, 5, 6 and 7 were informative, showing no maternal DNA in cord blood. SP4 was not informative at the YNZ22 locus. Overall, analyses of both D1S80 and YNZ22 successfully ruled out maternal DNA in cord blood of 35 (out for 37) SPs. **Gel C** shows one (SP8) of the two SPs that were not informative after genotyping of all three VNTR loci (D1S80, YNZ22 and ApoB).
(TIF)

S1 File Minimal dataset underlying main findings. (XLSX)

S1 Table Factors associated with the breadth of Pf IgM in cord plasma. (DOCX)

S2 Table Descriptive characteristics of the 64 Cameroonian newborns whose samples were used for *in vitro* antibody studies. (DOCX)
