# Supplementary figures and images for: Timing of the human prenatal antibody response to Plasmodium falciparum antigens
Source: PLoS One. 2017 Sep 26;12(9):e0184571. doi: 10.1371/journal.pone.0184571 (PMC5614534; doi:10.1371/journal.pone.0184571)

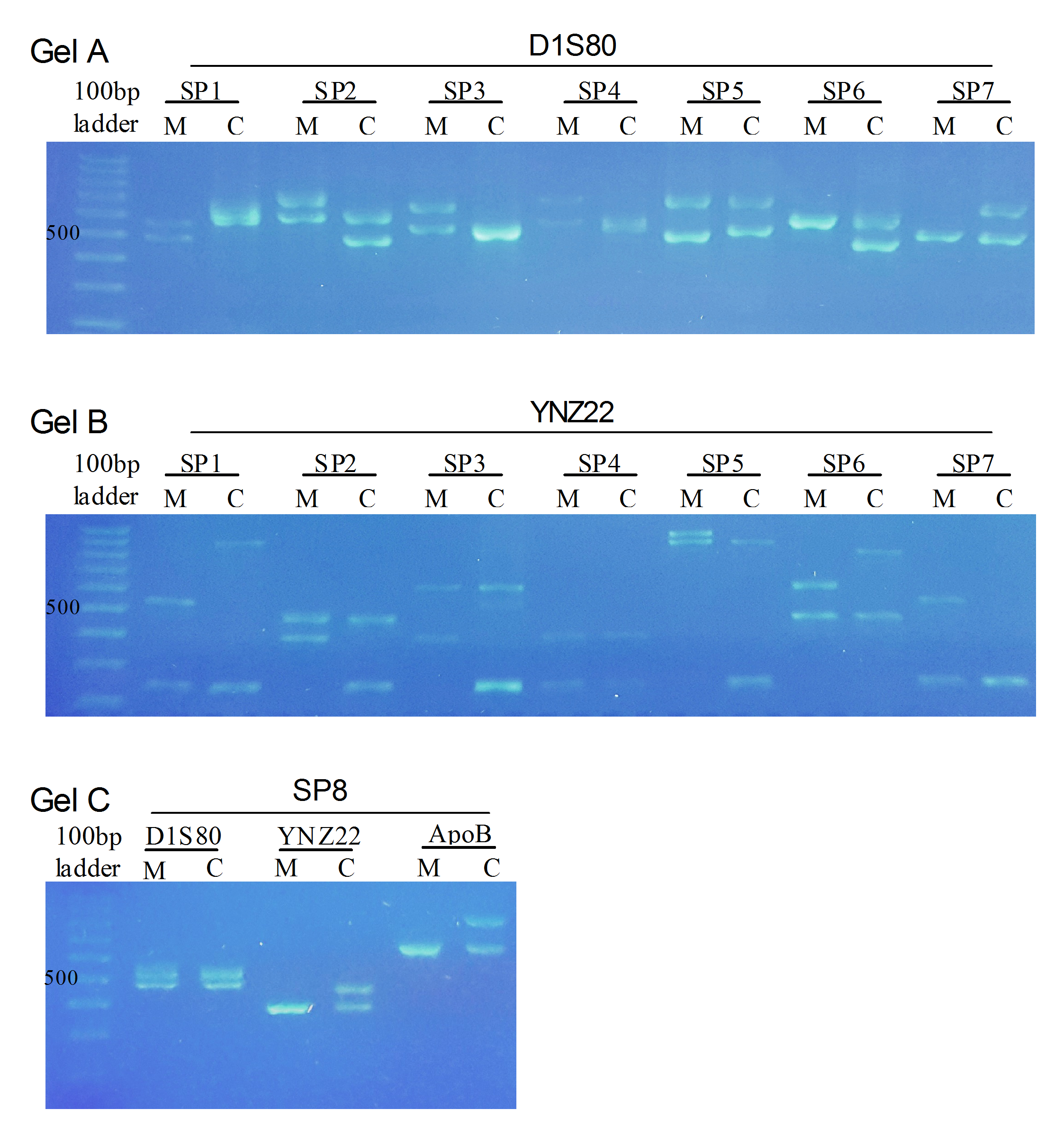

Supplement: S1 Fig — Bands represent peripheral maternal blood (M) and cord blood (C) VNTR alleles for mother-stillborn sample pairs (SP). Eight SPs (out of 37 SPs analyzed) are represented. Gel A shows D1S80 alleles in SP1through SP7. Maternal DNA was not detected in cord blood compartments of SP1, 2, 3, 4 and 5. SP6 and SP7 were not informative at the D1S80 locus since the mothers were homozygous. Gel B shows YNZ22 alleles for the same SPs. SP1, 2, 3, 5, 6 and 7 were informative, showing no maternal DNA in cord blood. SP4 was not informative at the YNZ22 locus. Overall, analyses of both D1S80 and YNZ22 successfully ruled out maternal DNA in cord blood of 35 (out for 37) SPs. Gel C shows one (SP8) of the two SPs that were not informative after genotyping of all three VNTR loci (D1S80, YNZ22 and ApoB). (TIF) [file pone.0184571.s001.tif]
