# Supplementary material for: Timing of the human prenatal antibody response to Plasmodium falciparum antigens
Source: PLoS One. 2017 Sep 26;12(9):e0184571. doi: 10.1371/journal.pone.0184571 (PMC5614534; doi:10.1371/journal.pone.0184571)
Supplement: S1 Table — (DOCX) [file pone.0184571.s003.docx]

**S1 Table. Factors associated with the breadth of Pf IgM in cord plasma**

| Factors | Number of Pf Ags recognized by Pf IgM responders | | P Value^a^ | Adjusted  P Value^b^ |
| --- | --- | --- | --- | --- |
|  | 1 or 2 Pf Ags | 3–5 Pf Ags |  |  |
| Number of deliveries | 123 | 46 |  |  |
| **Fetal factors** |  |  |  |  |
| Live births | 113 (92%) | 42 (91%) | 1.000 | 0.484 |
| Twin delivery | 7 (6%) | 0 (0%) | 0.192 | 0.987 |
| Birth weight in kg | 2.9 [2.1–3.4] | 3.0 [2.3–3.4] | 0.497 | 0.999 |
| Placental weight in kg | 0.6 [0.5–0.7] | 0.6 [0.5–0.7] | 0.555 | 0.750 |
| Preterm delivery^b^ | 58 (47%) | 17 (37%) | 0.297 | 0.277 |
| **Maternal factors** |  |  |  |  |
| Age in years | 27 [22–32] | 27 [20–32] | 0.695 | 0.500 |
| Primigravid women | 29 (24%) | 15 (33%) | 0.242 | 0.228 |
| Anemia | 34 (28%) | 23 (50%) | **0.003** | **0.001** |
| Malaria positive | 35 (28%) | 17 (37%) | 0.351 | 0.552 |
| Parasite density in % | 0.05 [0.01–0.73] | 0.10 [0.01–0.43] | 0.619 | 0.506 |

The analysis includes 169 Cameroonian newborns who had IgM to at least one of 5 Pf Ags (MSP1-[FVO or 3D7], MSP2-[FC27 or 3D7], AMA1, EBA175, RESA). ^a^Univariate analyses comparing medians [25^th^ – 75^th^ percentile] using the Mann-Whitney test or comparing number (column %) using Fisher’s exact test. ^b^Multivariate analyses by logistic regression including all variables in the table.
