# Supplementary material for: Timing of the human prenatal antibody response to Plasmodium falciparum antigens
Source: PLoS One. 2017 Sep 26;12(9):e0184571. doi: 10.1371/journal.pone.0184571 (PMC5614534; doi:10.1371/journal.pone.0184571)
Supplement: S2 Table — (DOCX) [file pone.0184571.s004.docx]

**S2 Table. Descriptive characteristics of the 64 Cameroonian newborns whose samples were used for *in vitro* antibody studies**

| Characteristics | Description |
| --- | --- |
| Number of newborns | 64 |
| Maternal characteristics |  |
| Age in years, median [25^th^ – 75^th^ perc] | 23 [20–29] |
| % Primigravid | 42.8% |
| % Malaria positive | 59.3% |
| Placental Pf density, median % iE [25^th^ – 75^th^ perc] | 0.7 [0 – 9.6] |
| Fetal characteristics |  |
| % Full term (≥37 weeks) | 100% |
| % Live births | 100% |
| % Singleton deliveries | 100% |
| Birth weight in kg, median [25^th^ – 75^th^ perc] | 3.1 [2.9–3.4] |

Pf, *Plasmodium falciparum*; iE, infected erythrocytes; perc, percentile; kg, kilograms
